# Neighbourhood delivery of urgent care in North Yorkshire, UK

**DOI:** 10.3399/bjgp26X744477

**Published:** 2026-03-01

**Authors:** Victoria Blake, Daniel Kimberling, Emma Olandj, Zulf Ali, Mike Holmes

**Affiliations:** 1 Nimbuscare, York, UK; 2 Haxby Group Practice, York, UK; 3 CEO York Medical Group, York, UK; 4 Chair, Trustee Board, RCGP, London, UK

## Introduction

Urgent care systems in England face increasing emergency department (ED) attendances and limited same-day primary care access, with general practice often operating at capacity. Evidence suggests that prolonged ED wait times are associated with increased mortality; patients waiting over 12 hours in ED have more than double the 30-day mortality risk compared to those seen within 2 hours.^1^

Recent NHS reports, including the Fuller Stocktake^2^ and the NHS 10 Year Plan^3^ sets out a clear direction for integrated, community-based care, describing how neighbourhood health models will be developed to improve access, strengthen prevention and continuity, and reduce pressure on hospitals. Evidence from neighbourhood urgent care innovations such as this now provides valuable insight into how those national ambitions can be delivered in practice.

In York, patients described the system in 2023 as ‘confusing’, ’complex’, and ‘difficult to navigate’, particularly in understanding the interface between GP practices, NHS 111, and emergency departments.^4^ Prior to April 2024 the GP out-of-hours (OOH) service, delivered by an independent private provider, faced significant staffing challenges and lacked digital integration with GP practices. This led to inefficiencies in triage, delayed call-backs, and reliance on alternative pathways such as ED. In April 2024 York and Scarborough Teaching Hospitals NHS Foundation Trust, as prime provider, awarded the GP OOH service contract to Nimbuscare, a local GP-led multi-neighbourhood provider.

To address longstanding challenges in urgent care pathways, Nimbuscare, in partnership with the Trust, implemented substantial improvements to the GP OOH service over a 12-month period. In December 2024, an in-hours same day urgent care (SDUC) model was introduced to improve access to GP appointments during the winter months. Designed for direct booking via general practice and delivered in each of York’s four neighbourhoods, this laid the foundation for North Yorkshire’s first fully integrated 24/7 urgent care service. This initiative aligns with national strategy and the local Integrated Urgent Care Service Specification,^5^ by supporting the shift towards community-based care and reducing reliance on acute services.

This article evaluates the implementation and outcomes of these service improvements, focusing on their impact on system utilisation, patient and provider experience, and cost-effectiveness, with the aim of informing future urgent care delivery models.

## What we did

We conducted a retrospective service evaluation of this novel integrated urgent care model comprising the redesigned GP OOH service and the in-hours SDUC service. We hypothesised that increasing capacity with bookable appointments from general practice and a responsive OOH service would decrease demand on downstream pathways such as NHS 111, urgent treatment centres (UTC) and EDs.

Models such as the Lincoln Clinical Assessment Service (CAS) informed the redesign of the GP OOH service in York and Scarborough.^6^ Commissioned by York and Scarborough Teaching Hospitals NHS Foundation Trust, the new service incorporated a programme of evidence-based quality improvements, evaluated over a 12-month period from April 2024. The SDUC model, was delivered between December 2024 and April 2025 to improve access to urgent care over winter. The timeline of key service developments in GP OOH and key features of the SDUC model are outlined in Table 1.

**Table 1.** Key features and developments of the redesigned urgent care model

Service	Intervention	Date	Key features / Developments
**GP Out-of- hours**	Clinical Assessment Service	April 2024	Co-located clinical and operational teams to manage NHS 111 calls
	Digital Integration	April 2024	Implementation of SystmOne, enabling access to full patient records and alignment with local GP digital systems
	Operational Dashboard	July 2024	Real-time monitoring of demand and capacity; staffing adjusted at intervals
	Community Frailty Hub Integration	August 2024	Co-located services enabling direct booking, reduced referral times, and safe transfers of care
**Same day urgent care**	Service Delivery	December 2024	Daily service 09:00–18:00 across York, Scarborough and Whitby
	Access	December 2024	Direct booking by GP practice reception using defined criteria
	Capacity	December 2024	Ringfenced appointments allocated by practice list size
	Digital Integration	December 2024	Use of SystmOne for consultation and care continuity
	Locations	December 2024	Delivered in each of York’s four Neighbourhoods. Appointments available in GP practices and urgent treatment centres co-located sites (York and Scarborough)
	Staffing	December 2024	GPs and advanced clinical practitioners with full admin support
	Appointment Structure	December 2024	15-minute consultation slots ensuring quality of care

Service data was obtained from SystmOne. NHS 111 call volume data was provided by the Yorkshire Ambulance Service and UTC (Type 3) attendance data was obtained from NHS England’s Analytical team. A cost analysis compared the SDUC cost-per-appointment with published estimates of urgent care costs from NHS England and the King’s Fund.^7^

## What we found

**Figure 1.**
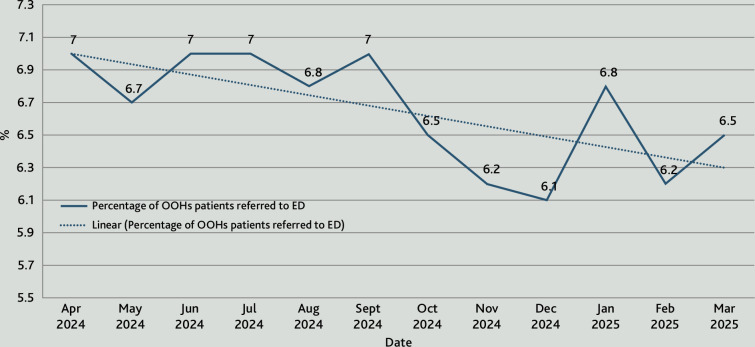
Monthly emergency department (ED) Referral Rate from the GP out of hours service (provided by Nimbuscare), 2024–2025. The chart shows a linear reduction in referral rates from a baseline of 7% to ∼6.3%. This represents ∼250 patients that were prevented from attending ED due to reduced referral rates from the GP out of hours (OOH) service.

**Figure 2.**
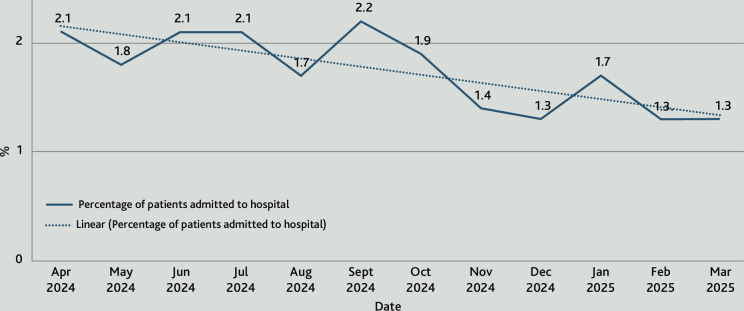
Monthly hospital admission rate from the GP out-of-hours service (provided by Nimbuscare), 2024–2025. The chart shows as linear reduction in referral rates for admission from a baseline of 2.1% to ∼1.3%. This represents ∼170 patients that were prevented from being admitted to hospital by the GP OOH service

Within the first 6 months of delivery, call-back times in the GP OOH service improved from 78% to 96% compliance with NHS 111 target timeframes. From April 2024 to April 2025 the service demonstrated a reduction of approximately 250 ED referrals and approximately 170 hospital admissions (Figure 1 and Figure 2).

**Figure 3.**
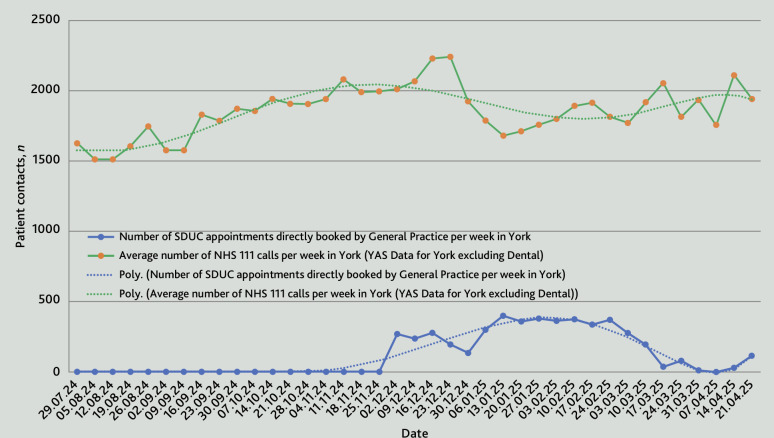
Relationship between the number of NHS 111 calls in York compared to the number of same day urgent care (SDUC) appointment booked by general practice in York from July 2024 to April 2025. NHS 111 call volumes increased steadily from July, peaking in December 2024. Following the implementation of the SDUC model, call volumes declined, before rising again after the service was decommissioned in April 2025

The SDUC model delivered over 8000 SDUC appointments across York, Scarborough and Whitby from December 2024 to April 2025, more than twice as many as the previous winter and with an average utilisation rate of appointments of 94%. During the delivery of SDUC, there was a corresponding reduction of a similar number of NHS 111 calls (Figure 3). Analysis of data from 2 December 2024 to 17 February 2025 demonstrates a statistically significant negative correlation between the two variables (Pearson’s r = –0.67, *P*=0.018). These findings suggest that the availability of SDUC appointments may have contributed to a reduction in NHS 111 call volumes.

In 2024 to 2025 there was a statistically significant reduction in the number of UTC (Type 3) attendances to York and Scarborough Teaching Hospitals NHS Foundation Trust compared to the previous year (Paired *t*-test=9.72, *P*=<0.05). The average reduction in UTC attendances across the Trust was >2100 patients per month (∼25 000 annually). This is most notable during the delivery of the SDUC appointments in York and Scarborough. We suggest that this reduction may be associated with the improvements in the GP OOH service from April 2024, alongside the effect of SDUC during winter 2024-2025.

General practice teams reported that the SDUC model provided valuable additional capacity during peak demand, was easy to book and well-coordinated. 87% of patients reported that their appointment met expectations. The total cost of the SDUC service was £134 000 resulting in a cost per appointment of £26. This compares favourably with other urgent care settings (ED attendance = £137–£445 and UTC attendance = £91).^7^

## What have you learned?

The SDUC model utilised non-recurrent funding and was decommissioned in April 2025 reflecting broader findings in the literature. Mason *et al* (2022) emphasised that integrated funding models can enable more efficient resource use and improved outcomes by aligning incentives.^8^ Their work supports the strategic shift of urgent care from hospitals into the community as a cost-effective alternative that delivers system-wide value. The SDUC model achieved cost savings compared to UTCs, by using existing clinical space, leveraging an established operational team, and staffing with a multidisciplinary mix of GPs and ACPs to minimise overheads. Future improvements could incorporate NHS 111 direct booking into SDUC, year-round delivery and strengthening referral pathways into Pharmacy First^9^ and Community Eye Care.^10^

The significant decline in UTC (Type 3) attendances to York and Scarborough Teaching Hospitals NHS Foundation Trust coincided with the implementation of the redesigned OOH service and was more pronounced during the delivery of the SDUC model. Notably, despite these clear improvements in urgent care delivery, the Trust’s performance against the 4-hour ED target did not significantly improve during this period. We suggest that this paradox may be partly due to a shift in case mix. Improvements in urgent care delivery may have diverted lower-acuity patients from hospital, leaving a higher proportion of complex cases that are harder to manage within the 4-hour window. As a result, performance metrics may underrepresent service improvements.

This issue highlights a disconnect between policy ambitions for integrated, community-based care and current hospital-centric reporting standards. The *Clinically-Led Review of NHS Access Standards* has noted that existing measures fail to account for patient acuity and system integration.^11^ As community-based urgent care models like these demonstrate effectiveness in reducing downstream pressure, new reporting structures are urgently needed to reflect true system improvement.

**Key Messages**

- Neighbourhood-based urgent care, led by GP multi-neighbourhood providers, can reduce reliance on hospitals and NHS 111.
- Delivering urgent care in community settings is more cost effective than urgent treatment centre and emergency departments attendances.
- Digital integration and shared clinical systems improve safety, responsiveness, and patient experience.
- Co-locating operational and clinical teams streamlines service delivery and enables operational and quality oversight.
- Sustained impact requires recurrent funding and performance measures that reflect system-wide improvement rather than single-provider metrics.
